# Difficulties in emotion regulation in patients with eating disorders

**DOI:** 10.1186/s40479-016-0037-1

**Published:** 2016-06-01

**Authors:** Catherine Ruscitti, Katrina Rufino, Natalie Goodwin, Rebecca Wagner

**Affiliations:** The Menninger Clinic, Baylor College of Medicine, Houston, USA; The Menninger Clinic – Compass Unit for Young Adults, 12301 Main Street, Houston, TX 77035 USA; University of Houston - Downtown, 1 Main Street, Houston, TX 77002 USA

**Keywords:** Eating disorder not otherwise specified, Eating disorder, Treatment, Inpatient, Emotion regulation

## Abstract

**Background:**

A defining characteristic of eating disorders (EDs) is difficulty with emotion regulation (ER). Previous research indicates that ED subtypes demonstrate differing ER difficulties. Specifically, individuals with Anorexia Nervosa (AN) or Bulimia Nervosa (BN) show greater impairment in their ability to regulate emotions in areas such as achieving goals while upset, reacting impulsively to distress, and effectively using coping strategies, as compared to those with Binge Eating Disorder (BED). However, limited research includes the diagnostic category of Eating Disorder, Not Otherwise Specified (EDNOS). The aim of this study was to better understand ER difficulties for all ED diagnoses, especially EDNOS. It was hypothesized that patients with EDs will demonstrate similar ER difficulties as psychiatric patients without EDs and that patients with EDNOS will be similar in their total level of ER difficulties but will differ in their specific types of difficulties in ER as compared to patients with other EDs.

**Methods:**

Participants included 404 adults presenting to an inpatient psychiatric hospital. Psychiatric diagnoses, including EDs, were determined using the Structured Clinical Interview for DSM Disorders. Differences in specific and overall difficulties with ER were examined across psychiatric patients using the multidimensional Difficulties in Emotion Regulation Scale.

**Results:**

Results of this study indicate that individuals with EDs have greater ER difficulties in most domains of ER and that those with BED and EDNOS demonstrate the most significant differences in ER as compared to psychiatric patients without EDs. Additionally, it was found that ED subtypes typically did not differ in terms of specific difficulties in ER. One exception emerged indicating that individuals with BED demonstrated significantly greater difficulty on the Limited Access to Emotion Regulation Strategies subscale as compared to those with EDNOS.

**Conclusions:**

Researchers were able to clarify difficulties in ER across ED diagnoses. Results highlight the importance of providing ER skills training for patients with EDs, particularly those with BED and EDNOS, and give insight into the specific areas of ER that may be important for these patients to focus on throughout recovery.

## Background

Eating disorders (EDs) are mental illnesses that affect individuals psychologically, emotionally, physically, and interpersonally. The overall prevalence of eating disorders is approximately 3.1 % for females, with Eating Disorder Not Otherwise Specified (EDNOS) representing the most common diagnosis in treatment settings, with prevalence rates around 2.4 % [16]. Eating disorders share many common features and patients may migrate from one ED diagnosis to another over the course of their illness [9]. As with many psychiatric disorders, one defining characteristic of EDs is difficulty with emotion regulation (ER; [20]). Patients with EDs have much greater ER difficulties compared to healthy and weight-matched controls (e.g., [4]), and interventions with an ER focus are beneficial to the treatment and outcomes of individuals with EDs [2, 5, 19].

Research has shown that varying ED symptom presentations and ED diagnostic classifications may indicate different types of difficulties with emotional regulation (e.g., [4, 7, 20]). In one study, Danner et al. [7] compared individuals with Anorexia Nervosa-Restricting subtype (AN-R), Anorexia Nervosa-Binge/Purge subtype (AN-BP), Bulimia Nervosa (BN), and Binge Eating Disorder (BED) using the Emotion Regulation Questionnaire (ERQ; [12]). Results indicated that individuals with AN-R and AN-BP scored significantly higher on Emotion Suppression than those with BN. Furthermore, those with BN scored significantly lower than those individuals with AN-BP on Cognitive Reappraisal.

Another study by Svaldi et al. [20] compared individuals with AN, BN, and BED using the Difficulties in Emotion Regulation Scale (DERS; [11]), the ERQ [12], and the Inventory of Cognitive Affect Reappraisal Strategies (ICARUS; [21]). While ED subtypes did not differ from each other on most of the variables researched, significant differences were found on the following five ER variables. On the ICARUS [21], individuals with AN were found to have significantly more difficulties than those with BED on Acceptance of Negative Situations and Reframing and Growth. Those with AN also had significantly more difficulties with Thoughts of Suicide than those with BN and BED. On the DERS [11], individuals with BN had significantly more difficulties than those with BED on Lack of Emotional Clarity. Additionally, those with BN and AN demonstrated significantly more difficulties than those with BED on Limited Access to Emotion Regulation Strategies.

Finally, Brockmeyer et al. [4] compared individuals with AN-R, AN-BP, BN, and BED using the DERS [11]. Results of this study indicated no differences between ED subtypes for Nonacceptance of emotional responses and Lack of Emotional Awareness. However, significant differences emerged for Lack of Emotional Clarity with individuals with AN-R, AN-BP, and BN demonstrating significantly more difficulties than those with BED. There was also a significant difference for Limited Access to Emotion Regulation Strategies, such that those with AN-BP demonstrated significantly more difficulties in this domain of ER than those with BED. On the Impulse Control Difficulties subscale, individuals with AN-BP demonstrated significantly more difficulties than those with AN-R and BED, and on the Difficulties in Engaging in Goal Directed Behavior subscale, individuals with BN reported significantly more difficulties than those with BED. Finally, in terms of Total scores, individuals with AN-BP and BN reported significantly higher levels of overall ER difficulties than those with BED.

While research has shown ER difficulties differ based on ED subtype, there exists limited research that includes patients with EDNOS in examining overall and specific ER difficulties as compared to patients with other ED diagnoses. Therefore, the purpose of this study was to: (1) Compare ER difficulties of patients at a general psychiatric hospital to previous research findings with regards to ER difficulties and ED diagnoses, (2) fill the gap in the literature by specifically examining ER difficulties in patients with EDNOS, and (3) examine specific ways in which difficulties in ER manifest in patients with EDNOS as compared to patients with other EDs.

We hypothesized that (1) given the transdiagnostic nature of ER difficulties across psychiatric disorders [20], patients with EDs will demonstrate similar ER difficulties as patients without EDs and (2) patients with EDNOS will demonstrate similar ER difficulties as psychiatric patients without EDs. Additionally, we predicted that (3) patients with EDNOS will be similar in their total level of ER difficulties as compared to patients with other EDs, and (4) patients with EDNOS will differ from patients with other ED subtypes in their specific difficulties in ER.

## Methods

### Participants

This study is part of a larger, hospital-wide outcomes study being completed at The Menninger Clinic, a private inpatient psychiatric hospital. Participants included patients diagnosed with a current or past ED based on diagnoses made from the Structured Clinical Interview for DSM Disorders (SCID; [10]), as well as comparison groups from the clinical population at Menninger. The study included archival data collected from July 2012 to December 2014. The present study included 404 participants ranging from 18 to 70 years of age (*M* = 31.26, *SD* = 12.71). The majority of the sample was female (*N* = 302, 74.8 %). A large majority (88.9 %) were Caucasian. The greatest number of participants reported completing some college (40.3 %), followed by a Bachelor’s degree (26.0 %), and a High School diploma (9.8 %). The average length of stay for the present inpatient sample was 50.14 days.

Of patients diagnosed with an ED, the majority met the criteria for EDNOS (*N* = 120, 62.8 %) followed by Anorexia Nervosa (*N* = 29, 15.2 %), Bulimia Nervosa (*N* = 22, 11.5 %), and Binge Eating Disorder (*N* = 20, 10.5 %). Among ED patients, the most frequent comorbid disorders were anxiety disorders (*N* = 132, 69.1 %), followed by substance use disorders (*N* = 121, 63.4 %) and depressive disorders (*N* = 115, 60.2 %). Among the non-ED patients, the most frequent diagnoses were depressive disorders (*N* = 136, 63.8 %), anxiety disorders (*N* = 131, 61.5 %), and substance use disorders (*N* = 117, 54.9 %).

### Measures

The Difficulties in Emotion Regulation Scale (DERS; [11]) is a 36-item scale used to measure ER difficulties. The DERS consists of six subscales: 1) Non-acceptance (six items), 2) Goals (five items), 3) Impulse (six items), 4) Awareness (six items), 5) Strategies (eight items), and 6) Clarity (five items). The Non-acceptance subscale evaluates one’s feelings about emotional responses, with higher scores reflecting negative feelings about emotional responses. The Goals subscale assesses the ability to accomplish goals in the midst of emotional states, with higher scores indicating greater difficulty accomplishing and concentrating on tasks when one is experiencing negative emotions. The Impulse subscale measures one’s ability to regulate behavior while under emotional distress, with higher scores indicating more impaired regulation. The Awareness subscale assesses the ability to attend to and acknowledge the significance of emotions, with higher scores reflecting lower awareness. The Strategies subscale evaluates one’s ability to influence emotional states, with higher scores indicating lower capacity to change how one feels. The Clarity subscale measures the extent to which individuals understand the emotions they are feeling, with higher scores indicating poorer understanding of feelings. Participants are asked to respond to each item using a five point Likert scale ranging from 1 (almost never) to 5 (almost always). Total and subscale scores are calculated by summing all the participants’ responses, with higher numbers reflecting greater difficulty with ER. The internal consistency of the DERS is 0.93 and all subscales of the DERS have internal consistencies greater than 0.80 [11]. Gratz and Roemer [11] also found adequate test re-test reliabilities between 0.57 and 0.89.

The Structured Clinical Interview for DSM-IV Diagnosis (SCID-I; [10]). The SCID-I was administered to all study participants to arrive at standardized, reliable diagnoses. The SCID was administered by two full time research interviewers, who were formally trained in administration and scoring, and who together have administered more than 1000 SCID interviews.

### Procedure

This study used archival data collected from The Menninger Outcomes Project. All adult patients admitted to the inpatient programs complete assessments online at admission, at 2-week intervals throughout their hospitalization, and at discharge. Patients were identified to participate in this project based on their having an ED diagnosis on the SCID, which was administered at admission. The scores from the patients’ DERS were also obtained upon admission. These individuals were matched to a group of similar peers for comparative purposes.

### Data analysis

For hypothesis 1 and 2, we used Propensity Score Matching (PSM) to match individuals from different groups based on a propensity score. By matching individuals on number of criteria met for Borderline Personality Disorder, the presence of a mood disorder, ethnicity, age, and gender, we were able to minimize these confounding variables. These variables were chosen in order to account for demographic information, as well as the ER difficulties associated with both Borderline Personality Disorder and mood disorders. Once matched, we conducted seven one-way ANOVAs, with each of the seven DERS scales (Total, Nonacceptance, Goals, Impulse, Clarity, Strategies, and Awareness; [11]) as dependent variables and ED diagnosis as the fixed factor.

For hypothesis 3 and 4, we conducted seven one-way ANCOVAs, with each of the seven DERS scales (Total, Nonacceptance, Goals, Impulse, Clarity, Strategies, and Awareness; [11]) as dependent variables and ED diagnosis as the fixed factor. The covariates included in the analyses were number of criteria met for Borderline Personality Disorder, the presence of a mood disorder, ethnicity, age, and gender. Bonferroni post hoc analyses were then conducted to examine specific group differences.

## Results

### Hypothesis 1: patients with EDs will demonstrate similar ER difficulties as psychiatric patients without EDs

One hundred ninety one patients with an ED diagnosis and 210 patients without an ED diagnosis who were matched via PSM were examined for this hypothesis using seven one-way ANOVA tests in order to compare the groups on the DERS Total score and each of the six DERS subscales. Results indicated that patients with an ED scored higher on all seven DERS scales than patients without an ED (see Table 1).

**Table 1:** Means and Standard Deviations for DERS Scores for Patients With and Without an ED Diagnosis

		Non-acceptance*		Goals*		Impulse		Awareness*		Strategies*		Clarity		Total DERS*	
Diagnosis	*N*	*M*	*SD*	*M*	*SD*	*M*	*SD*	*M*	*SD*	*M*	*SD*	*M*	*SD*	*M*	*SD*
ED	191	19.74	6.90	19.24	4.64	17.85	6.12	17.92	5.96	27.25	7.26	14.47	4.92	116.07	25.81
No ED	210	17.85	7.23	18.20	5.18	16.84	6.63	16.50	6.01	24.58	8.50	13.90	5.18	107.65	28.79
Total	401	18.75	7.13	18.69	4.95	17.32	6.41	17.17	6.02	25.85	8.04	14.17	5.06	111.66	27.70

*Significant group difference (*p* < 0.05)

The omnibus *F*-test was significant for the DERS Total, *F*(1, 399) = 9.44, *p* = 0.002, partial *η*^2^ = 0.023, indicating that there was a significant difference in DERS Total scores between patients with EDs and patients without EDs and that 2.3 % of the variance in DERS Total score was associated with diagnostic category, which represented a small effect. The omnibus *F*-test was also significant for the DERS Nonacceptance subscale, *F*(1, 399) = 7.18, *p* = 0.008, *partial η*^2^ = 0.018, the DERS Awareness subscale, *F*(1, 399) = 5.68, *p* = 0.018, *partial η*^2^ = 0.014, the DERS Goals subscale, *F*(1, 399) = 4.50, *p* = 0.035, *partial η*^2^ = 0.011, and the DERS Strategies subscale, *F*(1, 399) = 11.36, *p* = 0.001, *partial η*^2^ = 0.028, indicating that there were significant differences in Nonacceptance, Awareness, Goals, and Strategies scores between patients with EDs and patients without EDs and that each represented a small effect. No significant differences were found between the two groups for the Impulse and Clarity subscales.

### Hypothesis 2: patients with EDNOS will demonstrate similar ER difficulties as psychiatric patients without EDs

Because patients with EDNOS and BED were all diagnosed with EDNOS based on the DSM-IV-TR, we first combined these two groups and compared 137 patients with an EDNOS/BED diagnosis and 136 patients without an ED diagnosis who were matched via PSM. We then separated EDNOS and BED and compared 18 patients with a BED diagnosis and 18 patients without an ED diagnosis, as well as 119 patients with an EDNOS diagnosis and 118 patients without an ED diagnosis who were all matched via PSM. This hypothesis was examined with seven one-way ANOVA tests for each of the three comparisons in order to compare the groups on the DERS Total score and each of the six DERS subscales. Patients with EDNOS/BED, BED, and EDNOS scored higher on all DERS scales than patients without an ED diagnosis, except patients with EDNOS scored lower on the DERS Clarity subscale (see Table 2).

**Table 2:** Means and Standard Deviations for DERS Scores for Each Comparison Group

EDNOS/BED vs. No ED															
		Non-acceptance*		Goals		Impulse		Awareness*		Strategies*		Clarity		Total DERS*	
Diagnosis	*N*	*M*	*SD*	*M*	*SD*	*M*	*SD*	*M*	*SD*	*M*	*SD*	*M*	*SD*	*M*	*SD*
EDNOS/BED	137	19.76	6.94	19.28	4.59	17.89	5.94	18.12	6.19	27.49	7.42	14.37	4.90	116.75	25.70
No ED	136	17.90	7.05	18.31	5.01	17.08	6.64	16.48	5.95	25.00	8.44	13.88	5.21	108.32	29.03
Total	273	18.84	7.04	18.79	4.82	17.49	6.30	17.30	6.12	26.25	8.03	14.12	5.05	112.55	27.69
BED vs. No ED															
		Non-acceptance		Goals		Impulse		Awareness		Strategies*		Clarity*		Total DERS*	
Diagnosis	*N*	*M*	*SD*	*M*	*SD*	*M*	*SD*	*M*	*SD*	*M*	*SD*	*M*	*SD*	*M*	*SD*
BED	18	22.17	5.77	20.50	3.49	20.83	4.77	19.11	6.13	32.06	5.10	16.72	4.35	131.39	19.03
No ED	18	17.61	7.59	19.00	6.16	18.33	6.46	17.28	5.75	27.61	6.77	13.50	5.04	111.33	24.29
Total	36	19.89	7.03	19.75	4.99	19.58	5.74	18.19	5.93	29.83	6.33	15.11	4.92	121.36	23.79
EDNOS vs. No ED															
		Non-acceptance		Goals		Impulse		Awareness*		Strategies		Clarity		Total DERS	
Diagnosis	*N*	*M*	*SD*	*M*	*SD*	*M*	*SD*	*M*	*SD*	*M*	*SD*	*M*	*SD*	*M*	*SD*
EDNOS	119	19.40	7.05	19.09	4.72	17.45	5.99	17.97	6.22	26.80	7.49	14.01	4.89	114.54	25.92
No ED	118	18.44	7.15	18.39	4.94	17.05	6.47	16.14	6.25	25.36	8.51	14.05	5.23	108.85	29.35
Total	237	18.92	7.10	18.74	4.83	17.25	6.22	17.06	6.29	26.08	8.03	14.03	5.05	111.70	27.77
AN vs. No ED															
		Non-acceptance		Goals		Impulse		Awareness		Strategies		Clarity		Total DERS	
Diagnosis	*N*	*M*	*SD*	*M*	*SD*	*M*	*SD*	*M*	*SD*	*M*	*SD*	*M*	*SD*	*M*	*SD*
AN	28	19.54	6.45	18.79	5.07	17.07	6.86	17.07	4.90	27.07	6.21	14.61	5.13	112.11	25.55
No ED	27	18.22	7.36	18.00	4.84	17.59	6.42	17.78	5.53	25.59	7.49	15.15	5.19	110.67	26.94
Total	55	18.89	6.88	18.40	4.93	17.33	6.59	17.42	5.18	26.35	6.84	14.87	5.12	111.40	26.00
BN vs. No ED															
		Non-acceptance		Goals		Impulse		Awareness		Strategies		Clarity		Total DERS	
Diagnosis	*N*	*M*	*SD*	*M*	*SD*	*M*	*SD*	*M*	*SD*	*M*	*SD*	*M*	*SD*	*M*	*SD*
BN	21	18.95	7.30	19.29	4.74	18.43	7.06	16.81	5.69	25.76	7.44	14.10	4.77	113.33	27.04
No ED	21	17.05	7.18	17.29	5.18	16.19	7.53	15.81	6.49	24.62	9.74	13.05	5.50	104.00	29.26
Total	42	18.00	7.22	18.29	5.01	17.31	7.30	16.31	6.05	25.19	8.58	13.57	5.11	108.67	28.22

*Significant group difference (*p* < 0.05)

When comparing those with EDNOS/BED to those without an ED, the omnibus *F*-test for the DERS Total score was significant, *F*(1, 271) = 6.46, *p* = 0.012, *partial η*^2^ = 0.023, indicating that there was a significant difference in Total scores between patients with EDNOS/BED and patients without EDs and that 2.3 % of the variance in DERS Total score was associated with diagnostic category, which represented a small effect. The omnibus *F*-tests were also significant for the DERS Nonacceptance subscale, *F*(1, 271) = 4.80, *p* = 0.029, *partial η*^2^ = 0.017, the DERS Strategies subscale, *F*(1, 271) = 6.70, *p* = 0.010, *partial η*^2^ = 0.024, and the DERS Awareness subscale, *F*(1, 271) = 5.01, *p* = 0.026, *partial η*^2^ = 0.018, indicating that there were significant differences in Nonacceptance, Strategies, and Awareness scores between patients with EDNOS/BED and patients without EDs and that each represented a small effect. No significant differences were found between groups for the DERS Goals, Impulse, and Clarity subscales.

When comparing those with BED to those without an ED, the omnibus *F*-test for the DERS Total score was significant, *F*(1, 34) = 7.61, *p* = 0.009, *partial η*^2^ = 0.183, indicating that there was a significant difference in Total scores between patients with BED and patients without EDs and that 18.3 % of the variance in DERS Total score was associated with diagnostic category, which represented a large effect. The omnibus *F*-tests were also significant for the DERS Strategies subscale, *F*(1, 34) = 4.94, *p* = 0.033, *partial η*^2^ = 0.127, and the DERS Clarity subscale, *F*(1, 34) = 4.21, *p* = 0.048, *partial η*^2^ = 0.110, indicating that there were significant differences in Strategies and Clarity scores between patients with BED and patients without EDs and that represented a moderate to large effect. The omnibus *F*-test is approaching significance for the DERS Nonacceptance subscale, *F*(1, 34) = 4.11, *p* = 0.051, *partial η*^2^ = 0.108. No significant differences were found between groups for the DERS Awareness, Goals, and Impulse subscales.

When comparing those with EDNOS to those without an ED, the omnibus *F*-test for the DERS Awareness subscale was significant, *F*(1, 235) = 5.16, *p* = 0.024, *partial η*^2^ = 0.021, indicating that there was a significant difference in Awareness scores between patients with EDNOS and patients without EDs and that 2.1 % of the variance in DERS Awareness score was associated with diagnostic category, which represented a small effect. No other significant differences were found, indicating patients with EDNOS did not differ from patients without an ED on their DERS Total score or on the Impulse, Clarity, Nonacceptance, Goals, and Strategies subscales.

To be consistent with previous research, we also examined how patients with AN and BN compared to patients without an ED on their DERS scores. We compared 28 patients with an AN diagnosis and 27 patients without an ED diagnosis who were matched via PSM. We also compared 21 patients with a BN diagnosis and 21 patients without an ED diagnosis who were matched via PSM. Those with BN scored higher than those without an ED on each of the DERS scales and those with AN scored higher than those without an ED on DERS Total score, as well as the DERS Nonacceptance, Goals, and Strategies subscales (see Table 2). However, no significant differences were found on any of the DERS scales for either of the comparisons, indicating that patients with AN and BN did not differ from patients without an ED on their DERS Total score or on the DERS Nonacceptance, Goals, Strategies, Impulse, Awareness, and Clarity subscales.

### Hypothesis 3 and 4: patients with EDNOS will be similar in their total level of ER difficulties as compared to other EDs but they will differ from patients with other ED subtypes in their specific difficulties in ER

Researchers compared 28 patients with AN, 21 patients with BN, 18 patients with BED, and 119 patients with EDNOS. This hypothesis was examined with seven one-way ANCOVA tests in order to compare ED subtypes on the DERS Total score and each of the six DERS subscales controlling for age, gender, ethnicity, number of BPD criteria met, and presence of a mood disorder. The covariate, age, was significantly related to Total DERS scores, *F*(1, 177) = 4.87, *p* = 0.029, *partial η*^2^ = 0.027, the DERS Goals subscale, *F*(1, 177) = 4.23, *p* = 0.041, *partial η*^2^ = 0.023, and the DERS Strategies subscale, *F*(1, 177) = 4.66, *p* = 0.032, *partial η*^2^ = 0.026. The covariate, number of BPD criteria met, was significantly related to Total DERS scores, *F*(1, 177) = 22.33, *p* < 0.001, *partial η*^2^ = 0.112, the DERS Goals subscale, *F*(1, 177) = 9.79, *p* = 0.002, *partial η*^2^ = 0.052, the DERS Impulse subscale, *F*(1, 177) = 62.63, *p* < 0.001, *partial η*^2^ = 0.261, the DERS Strategies subscale, *F*(1, 177) = 15.25, *p* < 0.001, *partial η*^2^ = 0.079, and the DERS Clarity subscale, *F*(1, 177) = 14.11, *p* < 0.001, *partial η*^2^ = 0.074. The covariates, presence of a mood disorder, gender, and ethnicity, were not significantly related to the scores on any of the DERS scales.

The omnibus *F*-test for the DERS Strategies subscale was significant, *F*(3, 177) = 3.13, *p* = 0.027, *partial η*^2^ = 0.050, indicating that there was a significant effect of ED subtype on Strategies scores after controlling for age, gender, ethnicity, number of BPD criteria met, and presence of a mood disorder and that 5.0 % of the variance in DERS Strategies score was associated with diagnostic category, which represented a moderate effect. No significant differences were found between groups for the DERS Total score or the DERS Goals, Impulse, Awareness, Nonacceptance, and Clarity subscales. Post hoc comparisons were completed with a Bonferroni correction (with the family-wise error rate set to α = 0.05). There was a significant difference between the DERS Strategies score associated with individuals with BED and individuals with EDNOS (difference between means = 5.059, *p* = 0.022). No other pairwise comparisons between the groups were significant.

## Discussion

Overall, researchers were able to clarify difficulties in ER across ED diagnoses. Specifically, our first hypothesis that patients with EDs will demonstrate similar ER difficulties as psychiatric patients without EDs was not supported. In fact, patients with EDs demonstrated significantly more difficulty in terms of their overall ability to regulate emotions, their ability to accept emotional responses, their ability to accomplish goals in the midst of emotional states, their ability to attend to and acknowledge the significance of emotions, and their ability to influence emotional states. Our second hypothesis, that patients with EDNOS will demonstrate similar ER difficulties to psychiatric patients without EDs, was partially supported. Patients with EDNOS demonstrated similar scores on the DERS [11] as compared to those without EDs, except those with EDNOS demonstrated greater difficulty attending to and acknowledging the significance of emotions than those without EDs. This finding is consistent with previous research indicating that patients with EDs have higher levels of alexithymia and struggle more with identifying and communicating their feelings [17]. Our third hypothesis that patients with EDNOS will be similar in their total level of ER difficulties as compared to patients with other EDs was supported. Total scores on the DERS did not differ between ED subtypes, and thus patients across the ED spectrum demonstrated similar total levels of ER difficulties. Finally, our fourth hypothesis that patients with EDNOS will differ from patients with other ED subtypes in their specific difficulties in ER was partially supported. Generally, patients across the ED spectrum demonstrated similar ER difficulties, except those with BED demonstrated greater difficulty with their ability to influence emotional states than patients with EDNOS.

Our findings related to patients with BED are somewhat contradictory to previous studies that have found individuals with BED to demonstrate significantly lower levels of emotion dysregulation difficulties as compared to patients with AN or BN (e.g., [4]). However, patients with BED demonstrate significant levels of emotion dysregulation and negative mood, which increases rather than decreases following a binge episode [14]. In other words, the act of bingeing does not provide relief from emotional distress. On the other hand, individuals with AN and BN report a feeling of relief, and at times pleasurable emotions, as a result of restricting [18] and purging [1, 6]. Therefore, it makes sense that patients with BED would demonstrate a greater sense of difficulty in regulating their emotions as compared to psychiatric patients without EDs, as the main symptom of the ED does not provide the regulating effect that restricting and purging provide for individuals with AN and BN.

ER difficulties can be addressed through various treatment modalities and techniques and would be beneficial in treating patients with EDs. For example, Acceptance and Commitment Therapy (ACT; [13]) emphasizes acceptance and expansion to increase one’s ability to tolerate and manage emotions. ACT also focuses on identifying one’s values and using committed action to set and accomplish goals in the midst of emotional distress. Similarly, Dialectial Behavior Therapy (DBT; [15]) dedicates entire modules of learning to effectively tolerate distress and regulate one’s emotions. Cognitive Behavioral Therapy (CBT; [3]) emphasizes techniques such as cognitive reframing, relaxation, and systematic desensitization to help individuals manage and effectively influence their emotional states. Finally, the use of SMART Goals [8] and psychoeducation about emotions and how emotions relate to ED symptoms can be useful in helping patients increase their ability to accomplish goals and understand their emotional states.

There are several limitations to our study. First, data for this study was collected at a general inpatient psychiatric hospital where the majority of patients are admitted for psychiatric conditions other than EDs. Often, patients with primary AN, BN, or BED are referred to specialty ED facilities. Therefore, the majority admitting to the hospital with an ED fall in the EDNOS category. Consequently, the small sample sizes of patients with AN, BN, and BED likely decreased the power of the analyses. Future research should consider examining larger and equal sample sizes. Additionally, the SCID [10] was used to determine if patients had an ED and if so, which ED patients had. Because the SCID [10] is an interview that is done at the beginning of one’s hospitalization and for the purposes of research, the actual clinical diagnoses made by the patients’ individual treatment teams during their stay may differ. Finally, The Menninger Clinic is not a primary ED hospital, and therefore the patients who are diagnosed with an ED are often seeking treatment for psychiatric issues more primary than their ED. This may make generalizations to those with primary EDs difficult.

Future research may want to look further into the nuances of the EDNOS population, as patients with an EDNOS diagnosis can vary greatly in their presentation of symptoms. Thus, patients with the same diagnosis but with different symptomology may demonstrate differences in their difficulties with ER. It would also be useful to look at ER difficulties longitudinally to examine if ED subtypes differ in their improvement of ER over the course of treatment. Finally, the discrepancy in the results of our study and those from previous research involving the ER difficulties in patients with BED lends itself to further study.

## Conclusions

Patients with EDs demonstrate unique struggles with ER difficulties based on their ED diagnostic classification. Therefore, results of this study imply that in treating patients with EDs, it may prove beneficial to give additional emphasis and specifically focus on improving one’s ability to accept one’s emotional responses, to accomplish goals in the midst of distress, to be aware, attend to, and acknowledge one’s emotions and their significance, and to implement coping strategies to influence and manage emotions effectively. Our study also highlights the importance of identifying patients with BED, as they may require extra focus on learning effective coping mechanisms in dealing with their emotions and gaining insight and understanding the emotions they are feeling.

## Abbreviations

ER, emotion regulation; EDs, eating disorders.
